# Pathway Interaction Network Analysis Identifies Dysregulated Pathways in Human Monocytes Infected by* Listeria monocytogenes*

**DOI:** 10.1155/2017/3195348

**Published:** 2017-08-16

**Authors:** Wufeng Fan, Yuhan Zhou, Hao Li

**Affiliations:** ^1^Medical Department, Xiangyang No. 1 People's Hospital Affiliated to Hubei University of Medicine, Xiangyang, Hubei 441000, China; ^2^Department of Obstetrics and Gynecology, Xiangyang No. 1 People's Hospital Affiliated to Hubei University of Medicine, Xiangyang, Hubei 441000, China; ^3^Department of Laboratory Medicine, Xiangyang No. 1 People's Hospital Affiliated to Hubei University of Medicine, Xiangyang, Hubei 441000, China

## Abstract

In our study, we aimed to extract dysregulated pathways in human monocytes infected by* Listeria monocytogenes* (LM) based on pathway interaction network (PIN) which presented the functional dependency between pathways. After genes were aligned to the pathways, principal component analysis (PCA) was used to calculate the pathway activity for each pathway, followed by detecting seed pathway. A PIN was constructed based on gene expression profile, protein-protein interactions (PPIs), and cellular pathways. Identifying dysregulated pathways from the PIN was performed relying on seed pathway and classification accuracy. To evaluate whether the PIN method was feasible or not, we compared the introduced method with standard network centrality measures. The pathway of RNA polymerase II pretranscription events was selected as the seed pathway. Taking this seed pathway as start, one pathway set (9 dysregulated pathways) with AUC score of 1.00 was identified. Among the 5 hub pathways obtained using standard network centrality measures, 4 pathways were the common ones between the two methods. RNA polymerase II transcription and DNA replication owned a higher number of pathway genes and DEGs. These dysregulated pathways work together to influence the progression of LM infection, and they will be available as biomarkers to diagnose LM infection.

## 1. Introduction

*Listeria monocytogenes* (LM) is a gram-positive intracellular bacterial pathogen and it is observed in many habitats [1]. In human, the disease induced by LM is called listeriosis and is most common in immunocompromised hosts, newborns, pregnant mothers, and elderly individuals, with a mortality of 20–30% in these risk groups [2, 3]. In the European Union, listeriosis showed a rising trend that started in 2008, causing 2161 cases and 210 deaths in 2014, 16% more than in 2013 [4]. LM utilizes the cellular processes of host to influence cell-cell interactions, move intracellularly, and proliferate [5]. Much of this has been completed via applying the actin-based cytoskeleton of host [5] and by examining the alterations induced by pathogen in host cell signal transduction [6]. However, the effects of LM on host gene expression still remain poor. Thus, a comprehensive understanding of characterization of the LM-induced alterations in human gene expression will shed light on the molecular mechanisms underlying the disease process and yield biomarkers of clinical disease.

In the recent years, with the accumulation of large amount of “omics” data in public databases, gene expression profiles have been widely utilized to detect signatures. Frequently, many computational approaches have been created to identify differentially expressed genes (DEGs) between disease and normal conditions [7, 8]. Nevertheless, for the same disease, many of these DEGs extracted in one dataset are later observed not to work efficiently in another dataset [9]. Because of the poor performance ability of DEGs, several methods have been developed to detect potential pathogenic pathways which enhances the accuracy when the pathways are employed as biosignatures, relative to individual genes [10, 11]. Generally, the importance of pathways is measured by means of hypergeometric distribution [12], and pathways are analyzed independently [13]. It is worth noting that more than one pathway might be involved in a given disease, because of sophisticated nature of biological systems. Different pathways might have cross-talks with each other, and the dysregulation of one pathway may influence the activities of many related pathways. Hence, it is available to identify more reliable pathway signatures by considering the functional dependency or interaction between pathways. Significantly, protein-protein interactions (PPIs) form an overall interaction network which elaborates the global interaction among functionalities. In addition, network-based method has been widely applied to analyze interactions to further provide the insights into pathogenesis mechanism [14, 15]. Thus, we integrated pathway information and PPI network to construct a pathway interaction network (PIN) which considered the functional dependency between pathways [16]. Crucially, detecting dysregulated pathways will shed light on mechanisms of a given disease and provide clues for disease therapy [17, 18].

Herein, in the current study, we sought to extract dysregulated pathways based on the PIN. Specifically, gene expression profile of human peripheral monocytes infected by LM (accession number E-MEXP-1613) was recruited from the public database of EMBL-EBI. Cellular pathways and human PPIs were, respectively, obtained from the Reactome and String databases for further analysis. Then, after the genes were aligned to the pathways, principal component analysis (PCA) method [19] was used to calculate the pathway activity for each pathway based on the summary of the expression values of all genes in this pathway, and one seed pathway was detected based on the pathway activity scores. Afterwards, a PIN was constructed with each node standing for a cellular pathway on the basis of gene expression profile, PPIs, and cellular pathways. Finally, identifying dysregulated pathways from the PIN was performed according to the seed pathway and classification accuracy.

## 2. Materials and Methods

### 2.1. Gene Expression Profile and Data before Treatment

The gene expression profile under the series number of E-MEXP-1613 [20] was recruited from the EMBL-EBI database based on the platform of A-MEXP-162-Amersham CodeLink UniSet Human 10K I Bioarray. A total of 40 samples were obtained from five probands in the data profile E-MEXP-1613, including data from monocytes infected with LM (*n* = 10),* Staphylococcus aureus* (*n* = 10),* Streptococcus pneumoniae* (*n* = 10), and control samples without infection by bacteria (*n* = 10). In our study, with the goal of investigating the response in human monocytes to infection with LM, we only selected peripheral blood monocytes infected by LM and controls to do further analysis.

Before analysis, data was log-2 transformed and normalized using quantile approach [21]. After the probes were aligned to the gene symbols, the final gene expression matrix including 4369 genes was created. Then, the expression values of all genes in expression matrix were standardized based on the following equation.

(1) $$\begin{array}{c} z_{mn}=\frac{g_{mn}-mean\left(g_{m}\right)}{std\left(g_{m}\right)}, \end{array}$$

in which *g*_*mn*_ represented the expression value of gene *m* in sample *n* and mean(*g*_*m*_) and std(*g*_*m*_), respectively, stood for mean and standard deviation of the expression vector for gene *m* across all samples.

### 2.2. Preparation of PPIs and Cellular Pathways

All human PPIs were recruited from the String database [22]. String database includes manually curated protein interactions and uses confidence scoring to give an estimate of how likely an association is to occur. In our study, the global PPI dataset containing 787896 interactions among 16730 unique human proteins was obtained. Then, in order to minimize the ambiguity, only interactions with confidence score > 0.2 in the global PPIs were selected to construct the background PPIs. Next, we identified the common gene set between background PPIs and gene expression data. Finally, a new PPI set including 58015 interactions among 3897 genes was extracted for subsequent analysis.

Moreover, the predefined cellular pathways (1675 pathways) were downloaded from the Reactome database [23]. After that, the intersection of the genes in each defined pathway with microarray profile was extracted. Subsequently, a set of informative pathways were obtained for subsequent analysis after discarding pathways with gene size less than 5 or more than 100. To our knowledge, pathways having too few genes might not have enough biological information, and pathways owning too many genes might be too generic [24]. Overall, 670 informative pathways were picked out.

### 2.3. Calculation of Pathway Activity

After the genes were aligned to cellular pathways, an activity score for each pathway was defined as the summary of the expression values of all genes of this biological pathway. Specifically, PCA method [19] was utilized to obtain the summary of expressions of all genes belonging to each pathway, which could availably describe the internal structure of high-dimension dataset by reserving the variance in the data while converting the data into low-dimension space. Briefly, for pathway *k* in sample *n*, the activity score *P*_*kn*_ was calculated based on the following formula:

(2) $$\begin{array}{c} P_{kn}=w_{1nk}z_{1nk}+w_{2nk}z_{2nk}+\cdots +w_{mnk}z_{mnk}. \end{array}$$

In this formula, *z*_*mnk*_ stood for the standardized expression value of gene *n* from pathway *k* in sample *m* and *w*_*mnk*_ represented the weight for *z*_*mnk*_. That was to say, the activity of each pathway was considered as the linear combination of the expressions of all genes in this pathway, and each pathway was believed as a meta-gene.

The first principal component from PCA especially was utilized as the activity score for the corresponding pathway herein. The pathways which had different activities between disease and control conditions were possibly associated with the disease. That was to say, the activity score for a given pathway among LM-infected samples and control subjects was different, and the difference demonstrated the correlation to LM infection. The greater the difference was, the more close the relevance of this pathway to the LM infection was. In our study, the pathway with maximum changes in activity scores between disease and control conditions was selected as the seed pathway.

### 2.4. Identification of DEGs and Construction of PIN

In the present study, Student's *t*-test was employed to determine which genes were differentially expressed between the two groups using the criteria of *P* < 0.05. Moreover, we also computed Pearson's correlation coefficient (PCC) and the absolute value of PCC for the PPI interactions in these two groups.

A PIN was built with each node denoting a pathway, where one edge was laid between two pathways if they shared at least one gene or there were interactions between genes from the two pathways based on PPIs. Due to the condition specificity of gene expression and pathway activity, we further needed that at least one of the common genes between two pathways was differentially expressed in the two conditions, or the two genes that coded a pair of interacting proteins employed to lay an edge between two pathways were highly coexpressed (PCC absolute value > 0.8). If not, the edges between two pathways were discarded. As we all know, PCC, as a common measure, was used to measure the strength of the association between two variables [25]. In our study, the weight score for a pathway-pathway interaction was determined as the total |PCC| values of all genes. Hence, an original PIN was constructed. With the goal of understanding the cooperation of different pathways more fully, we simplified the original PIN mentioned above. The score values of each pathway interactions in the PIN, for example, summation of the absolute values of PCC for the PPIs in every two pathways, were computed. Then, the top 5% pathway interactions were selected to build a new PIN for the detection of dysregulated pathways.

### 2.5. Detection of Dysregulated Pathways from the New PIN

Specifically, a single pathway that could best discriminate between disease and control was firstly extracted as the seed pathway (the first pathway biosignature), and the second pathway that could be added to the first pathway to obtain better classification performance was selected from those pathways that interacted with the first pathway in the PIN. This process was repeated to add new pathways to detect pathway biomarkers till no more pathways could be added to improve classification accuracy, and the final selected pathway sets were regarded as potential dysregulated pathways in diseases.

In the selection procedure, support vector machines (SVMs) were utilized to formulate the detection of dysregulated pathways. The classification performance was evaluated using fivefold cross-validation, and Area Under The Curve (AUC) score was adopted as classification performance index. In an attempt to obtain robust results, fivefold cross-validation was repeated for 100 times and the mean value of classification accuracy was used as the final result.

### 2.6. Centrality Analysis for the Original PIN to Identify Significant Pathways

Centrality measures are broadly utilized for analyzing the properties of network, which cover degree, closeness, betweenness, and eigenvector centrality [26]. Among these parameters, degree is the simplest index. As documented, the definition of degree is the number of links that one node links with other nodes [27]. The degree centrality of the original PIN was analyzed. In the current work, the pathway nodes with degrees > 100 were identified as hub pathways.

## 3. Results

### 3.1. Construction of PIN

**Figure 1:**
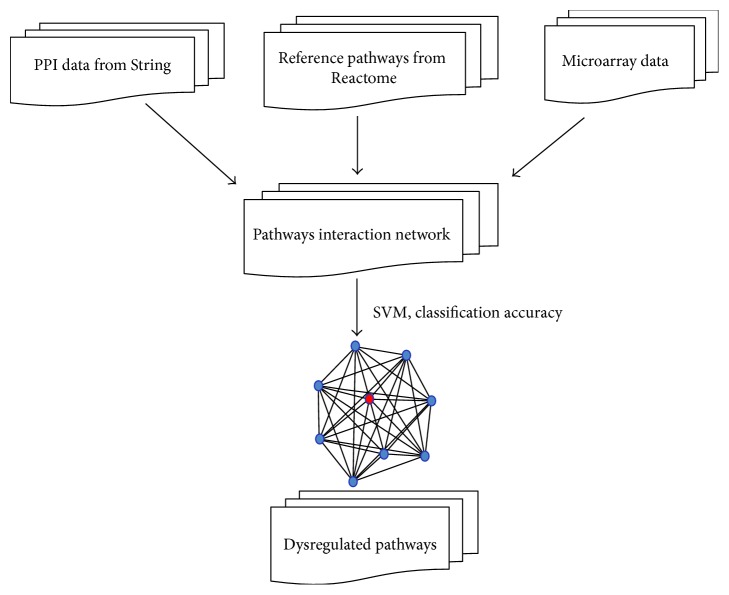
Workflow of detection of dysregulated pathways in LM-infected samples. Specifically, gene expression profile of human peripheral monocytes infected by LM (accession number E-MEXP-1613), cellular pathways, and human PPIs were, respectively, obtained from the corresponding databases. The PIN was constructed with each node standing for a cellular pathway on the basis of gene expression profile, PPIs, and cellular pathways. Finally, identifying dysregulated pathways from PIN was performed according to seed pathway and classification accuracy. The red node represented the firstly identified pathway called seed pathway, and the blue ones were those pathway markers that were combined with the seed pathway to obtain best classification accuracy while discriminating between LM-infected samples and controls.

**Figure 2:**
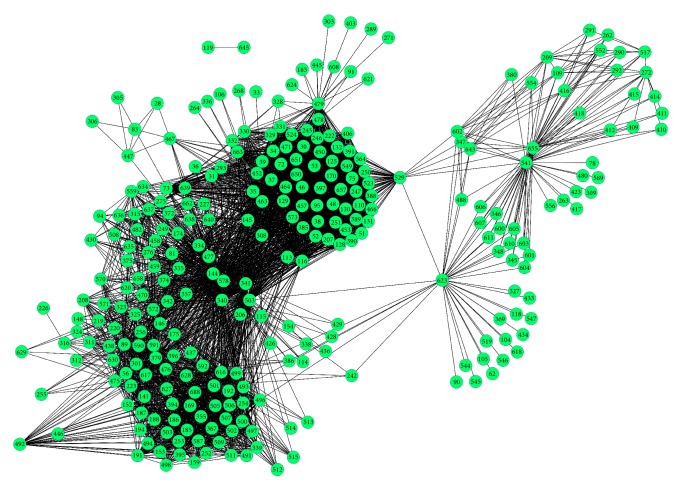
Pathway interaction network (PIN) for LM-infected samples. Nodes were on behalf of pathways and edges stood for the interaction between any two pathways.

The schematic diagram of detecting dysregulated pathways is shown in Figure 1. Based on the *P* < 0.05, a total of 1682 DEGs were selected. To construct the PIN, we conducted the selection for the edges between every two pathways based on the criteria of that at least one of the common genes between two pathways was differentially expressed in the two conditions, or the two genes that coded a pair of interacting proteins employed to lay an edge between two pathways were highly coexpressed (PCC absolute value > 0.8). Finally, an original PIN including 96270 interactions among the pathways was constructed. After we computed the summation of the absolute values of PCC for the PPIs in every two pathways, the top 5% pathway interactions were selected to build a new PIN for the detection of dysregulated pathways. Overall, a total of 4814 interactions were extracted to construct the new PIN, as shown in Figure 2. From this figure, we observed that pathways interacted with each other, but the strengths were different. The weight score for a pathway-pathway interaction was determined as the total |PCC| scores of all genes, and interactions with higher weight scores might be more important for LM-infected group than the others. The weight scores ranged from 25 to 135 among 4814 interactions. Interestingly, we found that only 9 pathway interactions owned the score values greater than 100. Among these 9 pathway interactions, the pathway of RNA polymerase II transcription (ID = 503) interacted with four pathways including nucleotide excision repair (ID = 379), RNA polymerase I (ID = 499), mRNA splicing (ID = 340), and mRNA splicing-major pathway (ID = 341). Specific information is shown in Table 1.

### 3.2. Identification of Dysregulated Pathways

Since there were differences for pathways in the new PIN, thereby how to assess the importance of each pathway and choose a significant one of the PIN became a challenge. An activity score was assigned to each pathway according to PCA method with the goal of evaluating its significance. The pathway of activity score with maximum changes between LM-infected samples and control conditions was selected to be seed pathway. In the current study, the seed pathway was RNA polymerase II pretranscription events (ID = 501). Taking this seed pathway as start, we conducted the identification of dysregulated pathways based on the classification accuracy increase. Overall, we obtained one pathway set (including 9 dysregulated pathways) with AUC score of 1.00. The good performance of this method demonstrated that the identified dysregulated pathways could be served as robust biomarkers. The detailed results are shown in Table 2. Of note, the pathway of RNA polymerase II transcription (ID = 503) had the maximum genes of 51, and the pathway of DNA replication (ID = 144) owned a higher number of genes of 44. More importantly, the pathway of DNA replication (ID = 144) and the pathway of RNA polymerase II transcription (ID = 503) had the most number of DEGs with 24 and the second highest number of DEGs with 23, respectively. The DEGs enriched in the final dysregulated pathways are shown in Supplemental Table 1 available online at https://doi.org/10.1155/2017/3195348.

**Figure 3:**
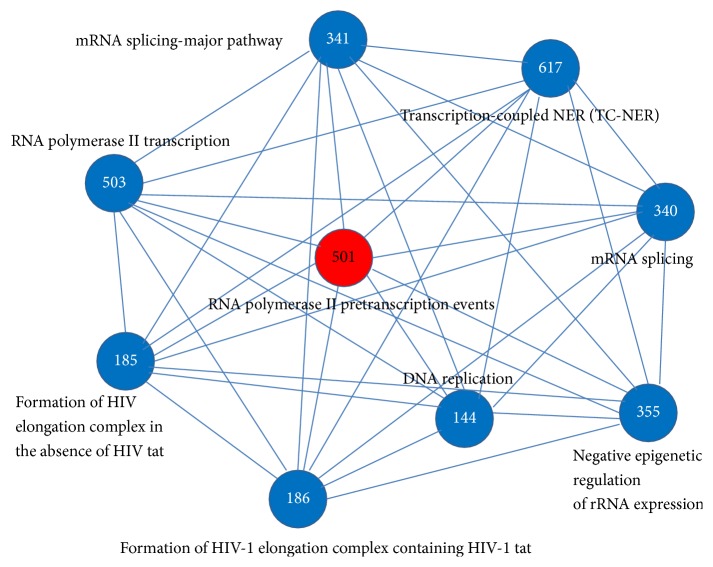
Dysregulated pathways interaction network (PIN). A total of 9 dysregulated pathways were screened out, which were assembled into a network based on their interactions in the PIN. Each node represented one dysregulated pathway. The red node stood for the seed pathway. The blue nodes were on the basis of the pathways that can be combined with the seed pathway to obtain best classification performance when discriminating between diseases and controls. The number stood for the pathway ID.

Figure 3 displayed the interactions among the 9 identified dysregulated pathways in PIN, where these 9 pathways had cross-talk with each other.

### 3.3. Topological Properties of the Original PIN

**Figure 4:**
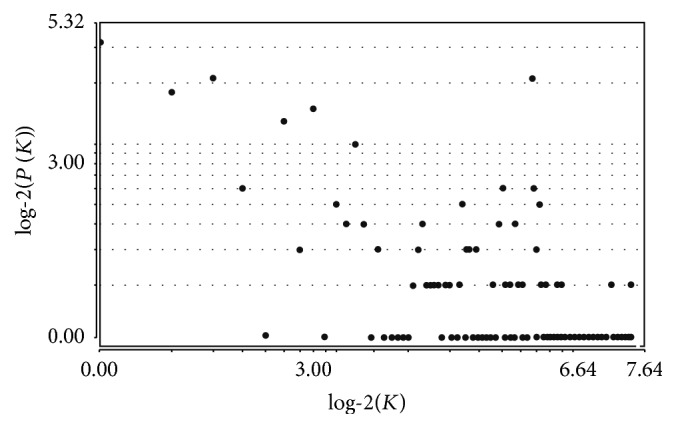
Degree distribution of all nodes in the original PIN. The *x*-axis represented log-2 based degrees, and the *y*-axis indicated the log-2 based frequencies of nodes with corresponding degrees.

With the goal of extracting the hub pathways in the original PIN, all nodes in the original PIN were ranked in a descending order based on the degree distribution of all nodes. The specific information of degree distribution is shown in Figure 4. Based on degrees > 100, a total of 5 hub pathways were identified, including DNA replication (ID = 144, degree = 176), synthesis of bile acids, and bile salts via 7alpha-hydroxycholesterol (ID = 578, degree = 176), RNA polymerase II transcription (ID = 503, degree = 169), mRNA splicing (ID = 340, degree = 146), and mRNA splicing- major pathway (ID = 341, degree = 146).

In order to evaluate whether the PIN method was feasible or not, we compared the introduced method with traditional topological analysis. We found that 4 hub pathways of DNA replication (ID = 144), RNA polymerase II transcription (ID = 503), mRNA splicing (ID = 340), and mRNA splicing-major pathway (ID = 341) were the common pathway obtained from PIN approach and topology method. Hence, we demonstrated that this PIN method can provide a flexible tool to extract pathway biomarkers for disease. Moreover, these identified dysregulated pathways will serve to benefit novel vaccine design and improve the therapeutic strategies in the infection procedure mediated by LM.

## 4. Discussion

LM is the etiology of listeriosis, which causes a severe human infection with 30% mortality [1]. Adaptive gene expression allows intracellular pathogens to successfully disseminate when encountering the immune defenses in the host cell. Nevertheless, the nature of the molecules that controls these processes is not well understood. Because LM has a facultative intracellular lifestyle, it is very crucially important to detect the biomarkers uniquely expressed intracellularly to further understand the infection processes and develop new strategies to limit listeria infections. Currently, pathway analyses have become the first option to expound the potential functions of genes, because it can enhance explanatory power [28]. Nevertheless, traditional pathway analyses mainly focused on single dysregulated pathway but did not consider the interactions among pathways [29]. Thus, we constructed the PIN which described the cross-talks among pathways.

In the current study, to obtain information towards identifying LM transcripts that correlate with infection of human monocytes, we used PCA method to calculate the activity value for each pathway, and the pathway of RNA polymerase II pretranscription events was selected as seed pathway. Eventually, we extracted 1 pathway set with AUC of 1.00 (9 dysregulated pathways), such as RNA polymerase II pretranscription events, DNA replication, and RNA polymerase II transcription. Among these 9 dysregulated pathways, the pathway of DNA replication and RNA polymerase II transcription had a higher number of pathway genes and DEGs. Moreover, the classification performance demonstrated the availability of this method to select dysregulated pathways in LM-infected samples and indicated that these dysregulated pathways were useful to be as biomarkers to diagnose disease.

Pathogen recognition and inducing immune reactions are crucial for efficiently counteracting infection. However, pathogen LM can use some strategies to avoid or modulate the immune detection. Several researches have demonstrated that LM manipulates the expression of host gene via modifying histones of immune genes which are activated by innate receptors during infection [30, 31]. Moreover, eukaryotic DNA is packed into chromatin, which is dependent on histones as well as chromatin-remodeling proteins [32]. Significantly, modification of histones has been indicated to cause the uncoiling of DNA exposing it to transcription factors [33]. More importantly, histone modifications and chromatin remodeling promote the regulation of eukaryotic gene transcription, thus regulating DNA replication, repair, or recombination [32]. As reported, LM has been indicated to replicate rapidly in the cytosol of host cells in the period of acute infection [34]. LM replication seems to represent a delicate balance between virulence factors and innate immune mechanisms of the infected cell. Once in the cytosol, LM replicates rapidly and usurps the host actin polymerization machinery to move through the cytosol and spread into neighbouring cells [34]. In our study, the pathway of DNA replication was the one pathway with the highest number of DEGs in the pathway set. Accordingly, we infer that LM exploits manipulating DNA replication to regulate the host response, thereby playing important roles in human infection.

In our study, the pathway of RNA polymerase II pretranscription events was regarded as the seed pathway. Moreover, RNA polymerase II transcription had the most number of DEGs with 24 in the pathway set. Type I interferons (IFNs) secreted by infected cells influence the development of innate and adaptive immune responses [35]. IFNs exerts deleterious functions in bacterial infections as well as autoimmune diseases [36]. A previous study has indicated that the magnitude of IFNAR signaling is suppressed by opposing mechanisms that limit the expression of IFNAR–JAK–STAT signaling components, and suppressive mechanisms include the pausing of RNA polymerase II at genes that encode IFN pathway components [37]. Accordingly, RNA polymerase II transcription might play important roles in LM infection process, partially via regulation of the IFN pathway.

Taken together, we successfully detected 1 pathway set with AUC of 1.00 (9 dysregulated pathways) in LM-infected human monocytes on the basis of the PIN which presented the functional dependency between pathways. The dysregulated pathways had cross-talks with each other. The functional relationship between pathways shed light on the molecular mechanisms of LM infection. Our data indicate that this created method is helpful to predict new biosignatures and even drug targets in robust way. Significantly, our identified dysregulated pathways will be available to be as biomarkers to diagnose LM infection, serve to benefit novel vaccine design, and improve the therapeutic strategies. However, several limitations must be taken into consideration. To begin with, there is a very high number of gene expression profiles concerning infection of diverse human cell lines with LM, but we only used E-MEXP-1613 dataset in our study. Thus, we will use other datasets about infection of diverse human cell lines with LM to verify our findings. Moreover, in the dataset of E-MEXP-1613, there was information about other bacterial infections including* Staphylococcus aureus* and* Streptococcus pneumoniae* infections. We did not compare the “pathway dysfunction” upon challenge with these other pathogens, and we would compare the specificity of the results obtained in this study in the future. Additionally, our analysis was implemented based on existing data using bioinformatics method; yet the findings have not been proved by animal experiments or patient tissue; this was the main weak point of this study. Therefore, further investigations are needed to uncover the changes of these pathways in the understanding of the infection procedure mediated by LM based on the animal experiments and patient tissues. Despite these limitations, this study provided some preliminary evidence to uncover alterative candidate therapeutic strategies for LM infection. Our analysis implies that this 1 pathway set (9 dysregulated pathways) worked together to influence the progression of LM infection, and making use of specific blockage-related pathways in LM infection will shed new insights for therapeutic and preventive methods in clinic.

## Supplementary Material

Supplemental Table 1: DEGs enriched in the final dys-regulated pathways.

## Figures and Tables

**Table 1:** The score distribution of the top 9 pathway interactions with scores > 100.

Pathway interactions	Scores
478:479	134.05899
379:503	104.345824
35:144	101.412355
144:463	101.412355
35:578	101.098052
463:578	101.098052
499:503	100.857179
340:503	100.525958
341:503	100.525958

*Note*. 478, respiratory electron transport; 479, respiratory electron transport; 379, nucleotide excision repair; 503, RNA polymerase II transcription; 35, APC/C-mediated degradation of cell cycle proteins; 144, DNA replication; 463, regulation of mitotic cell cycle; 578, synthesis of DNA; 499, RNA polymerase I; 340, mRNA splicing; 341, mRNA splicing-major pathway.

**Table 2:** Dysregulated pathways identified from the pathway interaction network (PIN).

ID	Pathways	Gene number in pathway	DEG number in pathway
501	RNA polymerase II pretranscription events	34	17

185	Formation of HIV elongation complex in the absence of HIV tat	23	13

186	Formation of HIV-1 elongation complex containing HIV-1 tat	23	13

617	Transcription-coupled NER (TC-NER)	26	10

355	Negative epigenetic regulation of rRNA expression	28	13

144	DNA replication	44	24

503	RNA polymerase II transcription	51	23

340	mRNA splicing	48	14

341	mRNA splicing, major pathway	48	14
