# Supplementary material for: Pathway Interaction Network Analysis Identifies Dysregulated Pathways in Human Monocytes Infected by Listeria monocytogenes
Source: Comput Math Methods Med. 2017 Aug 16;2017:3195348. doi: 10.1155/2017/3195348 (PMC5603742; doi:10.1155/2017/3195348)
Supplement: Supplementary file 1 — Supplemental Table 1: DEGs enriched in the final dys-regulated pathways. [file 3195348.f1.docx]

**Supplemental Table 1** DEGs enriched in the final dys-regulated pathways

| **Pathway index** | | **Pathway name** | | **DEGs** |  |  | |  | |  | |  | |  | |  |  |  |  | |  |  |  |  | |  |  |  |  |  |  |  |  |
| --- | --- | --- | --- | --- | --- | --- | --- | --- | --- | --- | --- | --- | --- | --- | --- | --- | --- | --- | --- | --- | --- | --- | --- | --- | --- | --- | --- | --- | --- | --- | --- | --- | --- |
| 501 | RNA Polymerase II pre-transcription events | | CCNH | | CTDP1 | | GTF2E1 | | GTF2E2 | | GTF2F2 | | GTF2H3 | | GTF2H4 | | | | | MNAT1 | | | | | NELFA |  |  |  |  |  |  |  |  |
|  |  | | NELFB | | POLR2H | | SUPT16H | | SUPT4H1 | | TAF11 | | TAF13 | | TCEA1 | | | | | TCEB3 | | | | |  |  |  |  |  |  |  |  |  |
|  |  | |  | |  | |  | |  | |  | |  | |  | | | | |  | | | | |  |  |  |  |  |  |  |  |  |
| 185 | Formation of HIV elongation complex in the absence of HIV tat | | CCNH | | CTDP1 | | GTF2F2 | | GTF2H3 | | GTF2H4 | | MNAT1 | | NELFA | | | | |  | | | | |  |  |  |  |  |  |  |  |  |
|  |  | | POLR2H | | SUPT16H | | SUPT4H1 | | TCEA1 | | TCEB3 | | NELFB | |  | | | | |  | | | | |  |  |  |  |  |  |  |  |  |
|  |  | |  | |  | |  | |  | |  | |  | |  | | | | |  | | | | |  |  |  |  |  |  |  |  |  |
| 186 | Formation of HIV-1 elongation complex containing HIV-1 tat | | CCNH | | CTDP1 | | GTF2F2 | | GTF2H3 | | GTF2H4 | | MNAT1 | | NELFA | | | | |  | | | | |  |  |  |  |  |  |  |  |  |
|  |  | | NELFB | | POLR2H | | SUPT16H | | SUPT4H1 | | TCEA1 | | TCEB3 | |  | | | | |  | | | | |  |  |  |  |  |  |  |  |  |
|  |  | |  | |  | |  | |  | |  | |  | |  | | | | |  | | | | |  |  |  |  |  |  |  |  |  |
| 617 | Transcription-coupled NER (TC-NER) | | APEX1 | | CCNH | | GTF2H3 | | GTF2H4 | | LIG1 | |  | |  | | | | |  | | | | |  |  |  |  |  |  |  |  |  |
|  |  | | POLR2H | | RFC3 | | TCEA1 | | XAB2 | | MNAT1 | |  | |  | | | | |  | | | | |  |  |  |  |  |  |  |  |  |
|  |  | |  | |  | |  | |  | |  | |  | |  | | | | |  | | | | |  |  |  |  |  |  |  |  |  |
| 355 | Negative epigenetic regulation of rRNA expression | | CCNH | | DNMT3B | | GTF2H3 | | GTF2H4 | | HDAC1 | | MNAT1 | | POLR1A | | | | |  | | | | |  |  |  |  |  |  |  |  |  |
|  |  | | POLR2H | | SUV39H1 | | TAF1D | | TTF1 | | UBTF | | POLR1E | |  | | | | |  | | | | |  |  |  |  |  |  |  |  |  |
|  |  | |  | |  | |  | |  | |  | |  | |  | | | | |  | | | | |  |  |  |  |  |  |  |  |  |
| 144 | DNA replication | | APEX1 | | FEN1 | | GINS1 | | GINS2 | | LIG1 | | MCM6 | | ORC1 | | | | | ORC5 | | | | |  |  |  |  |  |  |  |  |  |
|  |  | | PSMA1 | | PSMA3 | | PSMA4 | | PSMA5 | | PSMA7 | | PSMB2 | | PSMB7 | | | | | PSMB9 | | | | |  |  |  |  |  |  |  |  |  |
|  |  | | PSMC1 | | PSMC6 | | PSMD14 | | PSMD2 | | PSMD4 | | PSMD6 | | PSMD7 | | | | | PSME1 | | | | | RFC3 |  |  |  |  |  |  |  |  |
|  |  | |  | |  | |  | |  | |  | |  | |  | | | | |  | | | | |  |  |  |  |  |  |  |  |  |
| 503 | RNA polymerase II transcription | | CCNH | | CLP1 | | CPSF3 | | CSTF3 | | CTDP1 | | GTF2E1 | | GTF2E2 | | | | | TAF13 | | | | |  |  |  |  |  |  |  |  |  |
|  |  | | GTF2F2 | | GTF2H3 | | GTF2H4 | | MAGOH | | MNAT1 | | NELFA | | NELFB | | | | | TCEA1 | | | | |  |  |  |  |  |  |  |  |  |
|  |  | | NUDT21 | | POLR2H | | SLBP | | SNRPD3 | | SUPT16H | | SUPT4H1 | | TAF11 | | | | | TCEB3 | | | | |  |  |  |  |  |  |  |  |  |
|  |  | |  | |  | |  | |  | |  | |  | |  | | | | |  | | | | |  |  |  |  |  |  |  |  |  |
| 340 | mRNA splicing | | CLP1 | | CPSF3 | | CSTF3 | | CWC15 | | GTF2F2 | | HNRNPR | | MAGOH | | | | |  | | | | |  |  |  |  |  |  |  |  |  |
|  |  | | NUDT21 | | POLR2H | | SF3B6 | | SNRNP40 | | SNRPA1 | | SNRPD3 | | YBX1 | | | | |  | | | | |  |  |  |  |  |  |  |  |  |
|  |  | |  | |  | |  | |  | |  | |  | |  | | | | |  | | | | |  |  |  |  |  |  |  |  |  |
| 341 | mRNA splicing-major pathway | | CLP1 | | CPSF3 | | CSTF3 | | CWC15 | | GTF2F2 | | HNRNPR | | MAGOH | | | | |  | | | | |  |  |  |  |  |  |  |  |  |
|  |  | | NUDT21 | | POLR2H | | SF3B6 | | SNRNP40 | | SNRPA1 | | SNRPD3 | | YBX1 | | | | |  | | | | |  |  |  |  |  |  |  |  |  |
|  |  | |  | |  | |  | |  | |  | |  | |  | | | | |  | | | | |  |  |  |  |  |  |  |  |  |
